# Fame

**Published:** 1858-11

**Authors:** 


### FAME

Is a "*vox et præterea nihil.*" NAPOLEON the First, in the zenith of his power, was astounded, when assured by the Great Mogul, or somebody else equally famous, that he had never heard of him before. An Invalid wrote us, the other day, and gave as a reason for it: "Your world-wide reputation induces me to apply to you for aid." Our correspondent is most tremendously mistaken. If DAVID, the son of JESSE, was here, with his famous sling, he could throw a pebble straight up the street, right through CYRUS FIELD'S front-door; and although *Irving Place* is called the "Regal Region," and its residents, therefore, supposed to be persons who have leisure for culture and acquiring information, we do not believe our fame has projected itself as far as the youthful shepherd's missile would have been carried by the force of the kingly champion's right arm: as proof, "Who is Dr. HALL? In the name of common sense, who is he? where is he? and what is he? I have just been all over the country, and whenever I took up a paper or a magazine, my eye was sure to fall on 'HALL'S Journal of Health;' papers of all 'pathies' and all sects, of every party I ever heard of, and as wide asunder as the poles, quote 'HALL'S Journal of Health.' I was born here in New York, grew up in New York, and in New York have done business to the present hour, and I never met him, saw him, or heard of him, and yet the name of his Journal stared me in the face whenever I opened a newspaper, all Summer: my dear fellow, will you please tell me who Dr. HALL is?" Such was the speech, and evidently earnest inquiry, of a gentleman night before last, when, entering our worthy publisher's book-store, his eye fell on a pile of "HALL'S Journal of Health," and with a slap of the hand coming down upon them, with the noise of a young thunder, he aspirated as above.

Some time ago, in the dusk of a Summer's evening, not a living creature in the house with us, except the cook and some crickets, we were trying to think of something funny to laugh at and revive our spirits, when the door opened, and in came a six-footer, a stranger, with wit enough to begin at once and tell what he wanted. We liked him immensely for that, and had at once "leisure" for him. "Being in New York for a night, for the first time in my life, I felt anxious to improve the opportunity, and see two men, if nobody else—the Editor of the 'Journal of Health,' and DIXON, of the 'Scalpel.' I've seen you, and now I'll go and hunt up DIXON;" and away he went. But we called him back, and had a good long talk, late into the night. We found him to be a man of a fine head, and finer heart, having a nobility of sentiment and a power of thought, not common to our kind; but he was exhausting himself in vain plans for human amelioration, by means of those impossible principles which men embrace who make not the Bible their polar-star, and who, instead of spending their time in active good-doing, waste both mind and body in bootless theorizing on the impracticable and the untried. Under these influences, the health of our new acquaintance was giving way. We counselled him to a different course, and bade him good night and God-speed. In this connection, we advise our readers to beware of vain thoughts, of conjectural plans of human improvement, of untried modes of elevation and reform. Take the old paths—theorize less—do more. Waste not time in suppositions and in experiments; but do good, in feeding the hungry, clothing the naked—meanwhile, educating the body to support itself by work, the mind to think, and the heart to Heaven-instilled "CHARITY."

